# Primary cardiac lymphoma in internal septum to left atrium with progressive heart failure in which cardiac magnetic resonance imaging led to the diagnosis: case report

**DOI:** 10.1016/j.jccase.2026.04.007

**Published:** 2026-05-07

**Authors:** Motohiro Shingu, Tatsuya Nishikawa, Masahiro Yamada, Yuya Ohga, Hibiki Kadohara, Yuto Osumi, Kenta Ishibashi, Mana Hiraishi, Mitsuo Kinugasa, Yasutaka Hirayama, Kinta Hatakeyama, Koichi Tamita

**Affiliations:** a Department of Cardiovascular Medicine, Akashi Medical Center, Akashi, Japan; b Department of Cardiovascular Medicine, Yodogawa Christian Hospital, Osaka, Japan; c Department of Pathology, National Cerebral and Cardiovascular Center, Suita, Japan

**Keywords:** Cardiac tumor, Primary cardiac lymphoma, Diffuse large B-cell lymphoma

## Abstract

Primary cardiac lymphoma is an uncommon malignancy that usually affects the right atrium. We report a rare case of diffuse large B-cell lymphoma involving the interatrial septum and posterior wall of the left atrium in a 74-year-old woman who presented with chest pain and complete atrioventricular block accompanied by therapy-resistant heart failure. Results using conventional diagnostic tools including echocardiography and computed tomography were unclear, but there was moderate pericardial effusion. Despite standard heart failure therapy, symptoms progressed, so we performed cardiac magnetic resonance imaging (CMR) to exclude myocarditis. Unexpectedly, CMR showed a mass that was infiltrating the interatrial septum and the adjacent left ventricular wall, but the coronary artery structure was basically unaffected. Gallium-67 scintigraphy confirmed cardiac localization, and surgical biopsy established the diagnosis of diffuse large B-cell lymphoma. The clinical course was complicated by progressive heart failure and sepsis, and the patient was transferred for hematologic treatment. This case highlights the diagnostic value of CMR in detecting atypical primary cardiac lymphoma and delineating myocardial infiltration. In patients with unexplained heart failure and conduction abnormalities, CMR should be promptly considered to guide appropriate management and to improve outcomes.

**Learning objective:**

Primary cardiac lymphoma may present with atypical localization, including the interatrial septum, the posterior wall of the left atrium, and the basal left ventricular septum, which could cause progressive heart failure and atrioventricular block. We found cardiac magnetic resonance imaging to be especially helpful in this case for detecting cardiac tumors and myocardial infiltration. Early consideration of cardiac malignancy in unexplained conduction disturbance and refractory heart failure was helpful in diagnosis and management of our patient.

## Introduction

The incidence of primary cardiac malignancy is reported to be 0.02–0.05% based on autopsy data [1]. The median survival period of cardiac malignant lymphoma is approximately 1 year and diagnosis in the early stages is important [2], [3]. Owing to easy access, imaging modalities for cardiac malignancy mainly consist of cardiac echocardiography and computed tomography (CT) [4]. However, if there is no suspicion of a cardiac tumor, it may be difficult to accurately evaluate tumors by echocardiography and CT. Cardiac magnetic resonance imaging (CMR) is reportedly useful in detection of cardiac malignancy [5]. Here, we report a case of primary cardiac diffuse large B-cell lymphoma with attention to the imaging evaluation and management.

## Case report

**Fig. 1:**
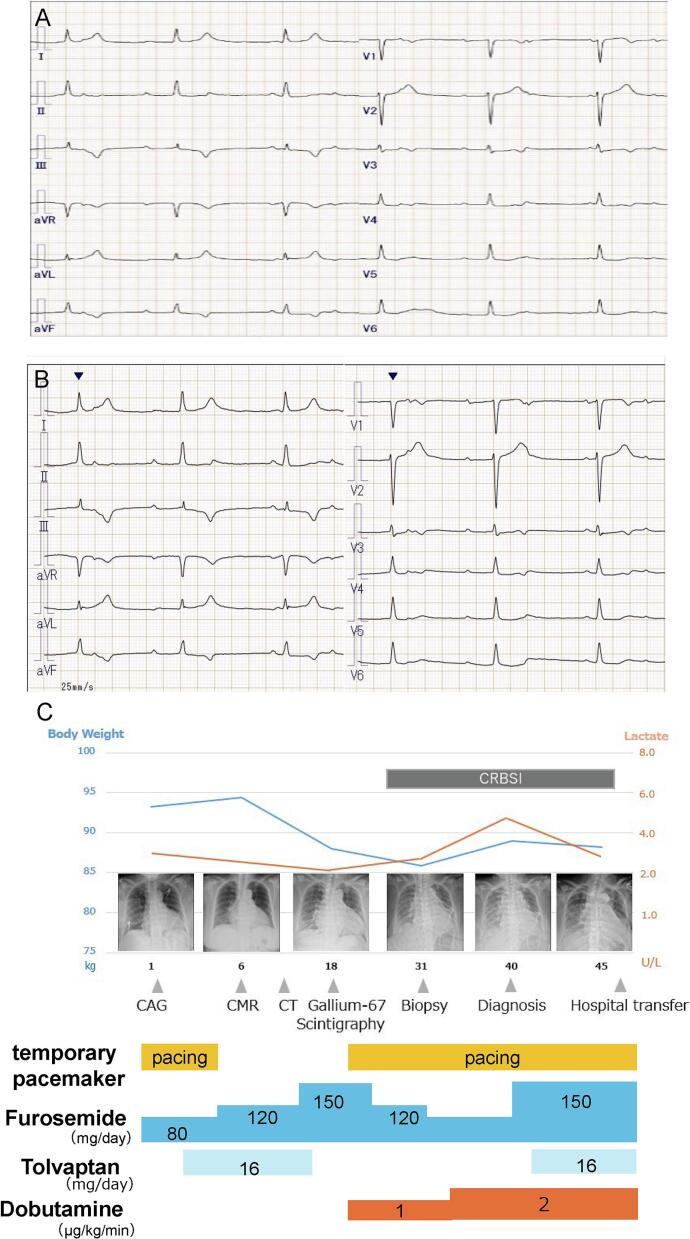
Electrocardiography and time course of clinical events. Electrocardiogram at admission (A) and immediately after temporary pacemaker removal (B) revealed complete atrioventricular block and that the junctional rhythm maintained a heart rate around 50 bpm. In echocardiography at admission, the findings of left atrial wall were unclear regarding whether there were any artifacts or structures. CRBSI, catheter-related bloodstream infection; CAG, coronary angiography; CMR, cardiac magnetic resonance imaging; CT, computed tomography.

**Fig. 2:**
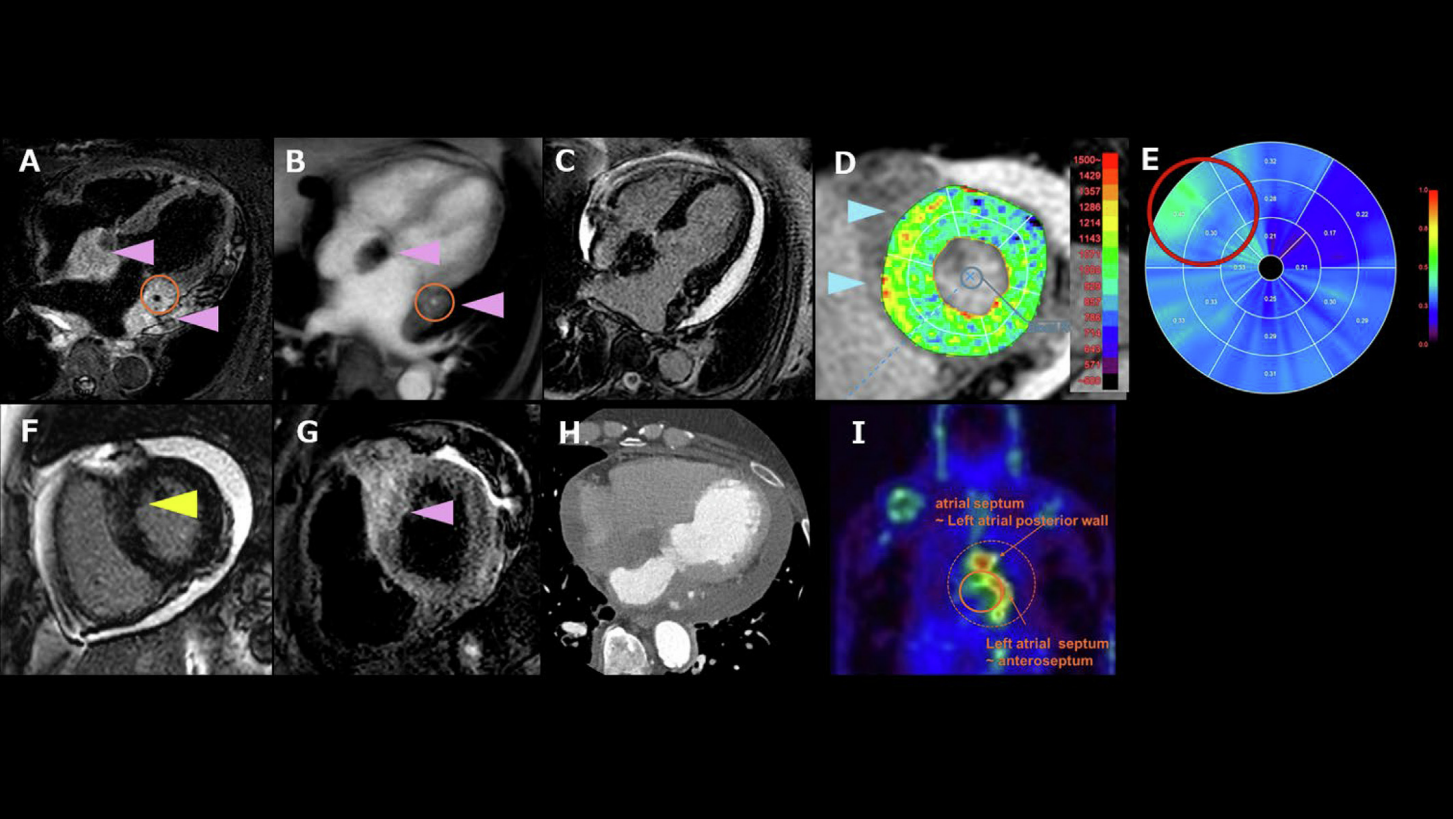
Cardiac Magnetic Resonance Imaging. (A) Black blood T2-short tau inversion recovery (T2-STIR) 4-chamber view. (B) Early enhancement of gadolinium. (C) Delayed enhancement of gadolinium, 4-chamber view. (D) Native T1 mapping in basal left ventricle. (E) Extracellular volume for all segments in left ventricle. (F) Delayed enhancement of gadolinium of basal left ventricle. (G) T2-STIR of basal left ventricle (H) Gallium-67 scintigraphy. (I) Contrast enhanced cardiac computed tomography. T2-STIR (A and G) revealed high signal intensity in the interatrial septum and left atrial wall and basal anteroseptal region of interventricular septum (purple arrowheads). The structure of the coronary arteries was preserved (A and I, orange circle). Delayed enhancement of gadolinium (F) showed the basal areas in left ventricular wall (yellow arrowhead) which was consistent with high native T1 (D: blue arrowhead), high extracellular volume fraction (E: red circle), and high intensity on T2- STIR (G). Gallium-67 scintigraphy (H) found no evidence of other sites of malignancy and showed accumulation in the mediastinum and left ventricle, which suggested left ventricular infiltration.

**Fig. 3:**
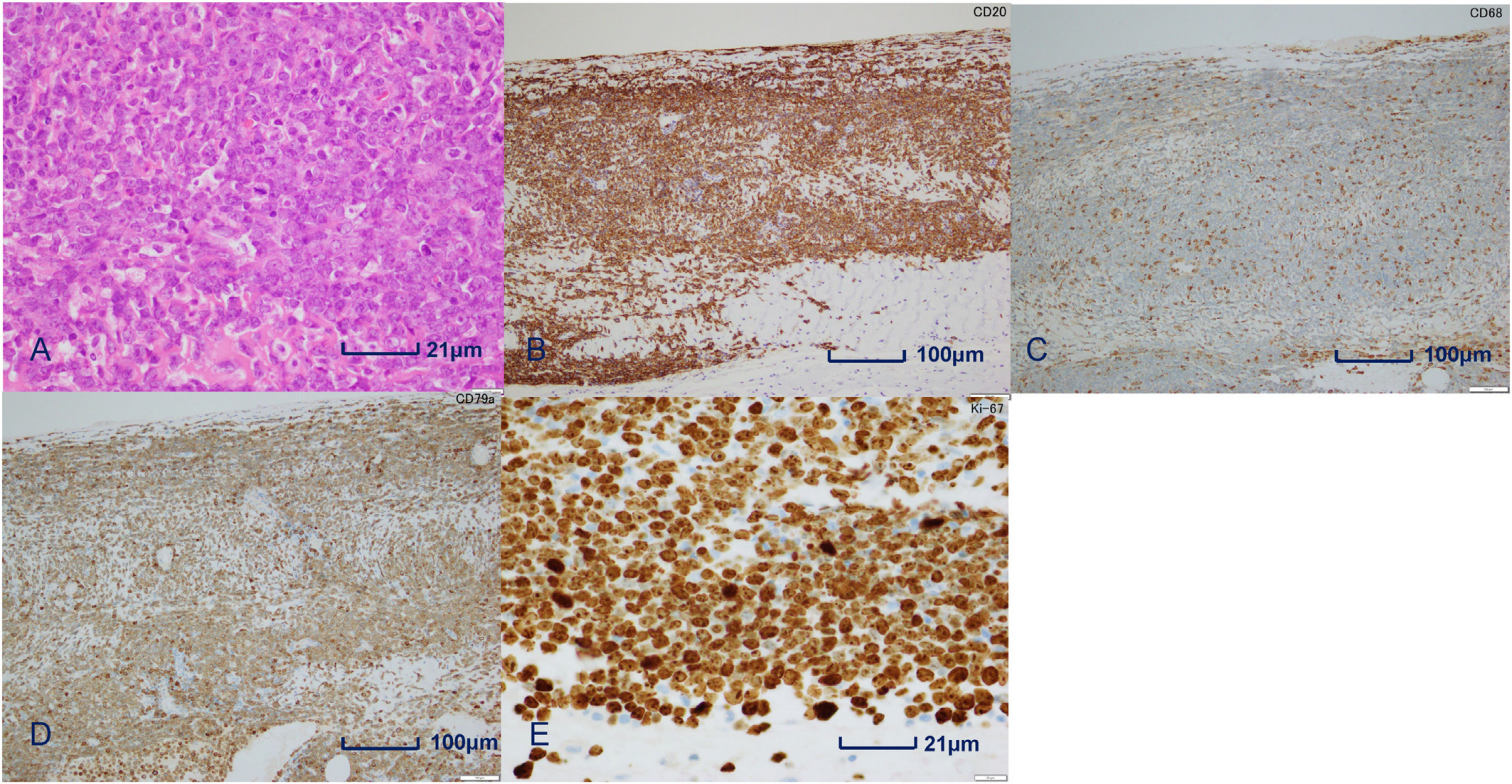
(A) Hematoxylin-eosin stain (HE stain). (B) CD20. (C) CD68. (D) CD79a. (E) Ki-67. HE stain shows diffuse invasion of large B cells. CD20 and CD79a are positive.

A 74-year-old woman presented with chest pain lasting for 3 h. Three months beforehand, there had been chest tightness at rest that persisted for around 10 min. Her medical history included hypertension, dyslipidemia, diabetes, chronic kidney disease, and old cerebral infarction. She had no history of smoking. On arrival, she was afebrile with blood pressure of 144/57 mmHg, a heart rate of 52 bpm, a respiratory rate of 22/min, and a SpO₂ of 97% (<2 L/min of nasal cannula). Her weight and height were 93.2 kg and 158 cm, respectively, and body mass index was 37.2 kg/m^2^. On admission, electrocardiogram showed complete atrioventricular block (CAVB) with a heart rate of 52 bpm (Fig. 1). Laboratory evaluation revealed the levels of creatine kinase 42 IU/L (normal range 106–211 IU/L), C-reactive protein 1.20 mg/dL (normal range 0.00–0.14 mg/dL), high lactate dehydrogenase 486 IU/L (normal range 124–222 IU/L), high troponin T 0.142 ng/mL (normal range < 0.014 ng/mL), and high N-terminal pro B-type natriuretic peptide 2428 pg/mL (normal range 0–125 pg/mL). Chest radiography showed pulmonary congestion. We performed transthoracic echocardiography (TTE) to evaluate the cause of CAVB and left heart failure. The patient was obese, and the images by TTE were poor (Video 1, Video 2). When retrospectively assessed, the atrial wall appeared to be thickened in four-chamber view, and a mass could be seen in the area between the left atrium and the aorta in transthoracic view (Video 1). However, at the point when TTE was taken, they were regarded as artifacts. Coronary angiography revealed no significant findings. The patient was managed with intravenous furosemide and noninvasive positive pressure ventilation for acute heart failure (HF). CAVB was considered to be a contributing factor of HF, so a temporary pacemaker was placed. On the third day, chest tightness remained unchanged, even when heart rate was maintained at 70 bpm with temporary pacing, and the patient could not be weaned off noninvasive positive pressure ventilation. We considered that CAVB might not be the main cause of the chest tightness and HF. Even without pacing, the patient's heart rate was maintained around 50 bpm with junctional rhythm, and blood pressure was stable. Furthermore, the symptoms and parameters of HF had not been improved by temporary pacing with higher rate in the clinical course. We removed the temporary pacemaker due to the risk of infection. At this point, we could have taken CMR under relatively stable hemodynamics. However, within a few days, the patient's chest pain worsened, and there were gradually rising lactate levels (Fig. 1). Considering the cause of CAVB and pericardial effusion, we performed CMR on the sixth day to rule out myocarditis (Fig. 1C). At this point, we had not suspected cardiac tumor as the main cause. The images of CMR clearly showed visually high intensity in the LA septum and posterior wall of LA in T2-short tau inversion recovery (STIR) (Fig. 2A). The CMR showed low-intensity lesion in early enhancement of gadolinium (Fig. 2B) although there was no clear finding in delayed enhancement of gadolinium (Fig. 2C). Furthermore, in the CMR, the basal septal areas in the left ventricle were thickened with visually high native T1 (local normal range, 973 ± 47) (Fig. 2D), and numerically high extracellular volume fraction (40%, Fig. 2E). In addition, delayed gadolinium enhancement (Fig. 2F) and high intensity in T2-STIR (Fig. 2G) was observed in the same area. The image findings met the criteria of diagnosis of myocarditis in the updated Lake Louise Criteria 2018 [6]. However, in the present case, first, we could detect the cardiac tumor in the interatrial wall and left atrium. Therefore, the adjacent basal left ventricular septal area was mimicking myocarditis, and actually it was infiltrated by tumor cells. We regarded the CAVB and worsening heart failure to have both originated from the cardiac tumor. Contrast-enhanced CT was useful because CT has high spatial resolution and ruled out a typical angiosarcoma according to the contrast in cardiac muscle. Furthermore, according to the characteristic that there was a mass around the coronary arteries with the structure of the coronary artery being preserved (Fig. 2H), the tumor was suspected to be lymphoma. We conducted gallium-67 scintigraphy to check for primary tumors throughout the body (Fig. 2I). It revealed uptake only in the heart, especially in inter atrioventricular and interventricular septum, and left atrioventricular wall, but no uptake was shown in other organs. The level of soluble interleukin-2 receptor was as high as 3270 U/mL (normal range 121–613 U/mL). To diagnose the type of cardiac tumor, surgical biopsy and pericardial window for refractory pericardial effusion was performed by cardiac surgeons on the 31st day. The pathological results were obtained on the 40th day, confirming a diagnosis of diffuse large B-cell lymphoma (Fig. 3). In the biopsy specimen, monotonous proliferation and infiltration of large, atypical lymphocytes were observed, and occasionally there were also mitotic figures. In immunohistochemical staining, tumor cells were positive for CD20 and CD79a (Fig. 3).

During treatment for HF, an increase in inflammatory response was gradually observed from around the 35th day, with lactate levels increasing on the 40th day. High levels of white blood cells (22,880/μL), C-reactive protein (4.29 mg/dL), and lactate (4.33 U/L) were observed. HF was thought to have worsened due to sepsis triggered by catheter-related bloodstream infection, so we started piperacillin/tazobactam and vancomycin with doses based on therapeutic drug monitoring. Regarding diffuse large B-cell lymphoma, it was necessary to involve the departments of hematology and oncology, and the patient was transferred to a higher-level medical facility to receive specialized treatment on 45th day.

## Discussion

Primary cardiac diffuse large B-cell lymphoma typically occurs in the right atrium and right ventricle [7]. In this unusual case, the malignant cardiac lymphoma was atypically located in the atrial septal wall and posterior wall. The lymphoma had a non-solid mass-like appearance, which made early diagnosis difficult. We found CMR to be especially useful for the diagnosis because T2-STIR clearly showed the cardiac tumor in the interatrial septum and the left atrium. Furthermore, with T1-mapping, T2-STIR, and extracellular volume fraction assessment, infiltration of lymphoma into the left ventricle could be detected. Additionally, CMR detected the intact coronary artery structure, which is reportedly a typical characteristic of cardiac lymphoma [8]. The key points in this case were the atypical clinical course of heart failure refractory to treatment, the poor echogenicity on the initial echocardiogram, and the rare diagnosis of diffuse large B-cell lymphoma. CMR played a key role in this diagnosis, and it was used to assess the severity of the cardiac tumor.

Primary cardiac diffuse large B-cell lymphoma can have a poor prognosis with survival of less than 1 month. However, with proper and early diagnosis and management, survival can be extended to 5 years [9]. In the present case study, the progressive HF was the most significant prognostic factor. The extensive progressive clinical course of HF was most likely due to infiltration of malignant cells into the left ventricle, as shown by gallium scintigraphy and CMR [10]. The HF was not sufficiently controlled because chemotherapy for lymphoma, the basic cause of the HF, had not been initiated.

Clinical symptoms of primary cardiac diffuse large B-cell lymphoma vary depending on the location of the tumor, but include congestive HF, pericardial fluid, and pleural effusion. In this case, the lymphoma developed in the atrioventricular node, which might have led to the CAVB, the area with highest intensity in gallium scintigraphy. In patients with acute HF with CAVB and progressive clinical course of HF, we suggest primary malignant cardiac lymphoma should be considered in differential diagnosis, and we recommend urgent therapeutic intervention, including management of CAVB.

## Conclusion

CMR has emerged for diagnosing primary cardiac tumors. In this case, we found it useful for reaching diagnosis of diffuse large B-cell lymphoma in the interatrial septum and left atrium and of the infiltration of the malignant cells into the left ventricle.

## Patient permission/consent statement

Identifying details of the present case were omitted to maintain anonymity.

## Declaration of competing interest

The authors declare that they have no conflict of interest.

## Appendix A Supplementary data

Video 1 Parasternal long-axis view in tansthoracic echocardiography

Video 2 Four-chamber view in tansthoracic echocardiography
